# AI-powered advances in type II endometrial cancer: global trends and African contexts

**DOI:** 10.3389/fonc.2025.1581645

**Published:** 2025-07-09

**Authors:** Thulo Molefi, Lloyd Mabonga, Rodney Hull, Motshedisi Sebitloane, Zodwa Dlamini

**Affiliations:** ^1^ Discipline of Obstetrics and Gynaecology, School of Clinical Medicine, University of KwaZulu-Natal, Durban, South Africa; ^2^ SAMRC Precision Oncology Research Unit (PORU), DSI/NRF SARChI Chair in Precision Oncology and Cancer Prevention (POCP), Pan African Research Institute (PACRI), University of Pretoria, Pretoria, South Africa; ^3^ Department of Medical Oncology, University of Pretoria, Pretoria, South Africa

**Keywords:** artificial intelligence, Type II endometrial cancer, African healthcare, personalized medicine, diagnostics, treatment planning, prognostic modeling

## Abstract

**Introduction:**

The advent of artificial intelligence (AI) in oncology has opened new avenues for enhancing the diagnosis, treatment, and prognosis of type II endometrial cancers (ECs), which account for the majority of EC-related deaths globally. With rising incidence and increasing concerns in Africa, type II ECs are often detected in advanced stages, exhibit aggressive progression, and resist conventional therapies. Despite these characteristics, they are still treated similarly to type I ECs, which are less aggressive and more treatment-responsive. Currently, no specific targeted therapies exist for type II ECs, creating an urgent need for innovative treatment options.

**Methods:**

This review examines the integration of AI-powered approaches in the care of type II ECs, focusing on their potential to address rising incidence and disparities in Africa. It explores AI-driven diagnostic tools, tailored therapeutic options, and ongoing innovative projects, including efforts to integrate indigenous knowledge into AI applications.

**Results:**

AI-powered therapeutic options tailored to the unique clinical profiles of type II EC patients show promise for developing targeted therapies. Several innovative projects are underway, leveraging AI to meet Africa’s unique healthcare challenges. These applications demonstrate significant potential to reduce healthcare disparities and improve patient outcomes, especially in resource-limited settings.

**Discussion:**

This review highlights the transformative potential of AI technologies in improving the diagnosis, treatment and management of type II ECs, particularly in Africa, where healthcare disparities are significant. Through the integration of AI in the type II EC care continuum, challenges in African healthcare can be overcome. Innovative projects, leveraging AI to meet the continent’s challenges, have the potential to improve patient outcomes. AI-driven therapies hold the key to personalized oncologic care, and indigenous African knowledge can be used to develop Afrocentric healthcare solutions. In Future, with continued research and the development of robust frameworks and transparent algorithms, investment and collaboration, the potential of AI in Type II EC will be realized.

## 1 Introduction

Artificial intelligence (AI) has emerged as a transformative force across various sectors, with profound impacts on healthcare, particularly in oncology (1). The use of AI-powered technologies in endometrial cancer (EC) research, diagnosis, treatment, and patient management marks a significant step forward in precision medicine (2). ECs are the most common malignancy of the female reproductive system, originating in the lining of the uterus (the endometrium) (3). They account for approximately 3% of all cancer diagnoses among women globally (2), with an estimated 417,000 new cases and more than 97,000 deaths reported annually since 2020 (4). ECs are divided into two primary categories based on their histopathological features: Type I and Type II (see **Table 1**) (5).

**Table 1:** A comparison between Type I and Type II endometrial cancers.

Feature	Type I EC	Type II EC
Prevalence	Approximately 80% of all EC cases	Less common, but more aggressive
Hormone Dependency	Estrogen-dependent	Not hormone-dependent
Tumour Grade	Low-grade endometrioid tumors	High-grade tumors
Prognosis	Favourable, with over 80% five-year survival rates	Poor prognosis, high propensity for rapid progression
Histological Subtypes	Mainly endometrioid tumors	Serous adenocarcinomas, clear cell adenocarcinomas, and carcinosarcomas
Detection Stage	Often detected at earlier stages	Often detected at advanced stages
Treatment Response	More responsive to conventional therapies	Often resistant to conventional therapies
Targeted Therapies	Currently available	No specific targeted therapies

Type I ECs, which make up approximately 80% of all cases, are typically estrogen-dependent and composed of low-grade endometrioid tumors. These tumors generally present a favorable prognosis, with over 80% five-year survival rates, attributed to their less aggressive behavior and detection at earlier stages (6). Conversely, Type II ECs are characterized by high-grade tumors with a high propensity for rapid progression and poor prognosis (5). This group mainly comprises three main histological subtypes: serous adenocarcinomas (accounting for 10–20%), clear cell adenocarcinomas (less than 5%), and carcinosarcomas (less than 5%) (7). Type II EC is often associated with specific histological subtypes (such as serous and clear cell carcinomas) and molecular markers such as TP53 mutations, HER2/neu amplification, and PI3K/AKT/mTOR pathway alterations, which are linked to aggressive tumor behavior, poorer prognosis, and limited hormonal responsiveness.

Regardless of the complexities around Type II ECs’ poorer prognosis, they are still being ineffectively treated in the same way as clinically indolent and readily curable type I ECs (5). Currently, there are no targeted therapies for Type II ECs. In this context, the integration of AI in Type II EC care has marked significant progress, offering innovative solutions in diagnostics, treatment planning, and prognosis (8). Globally, AI algorithms have enhanced the accuracy of histopathological image analysis, identifying biomarkers for early detection, and the development of personalized treatment strategies (9). By leveraging machine learning algorithms, big data analytics, and deep learning, AI systems can process vast amounts of clinical, imaging, and genomic data to aid in the early detection, enhancement of diagnostic accuracy, and personalize treatment strategies (1). High-income countries have made notable progress, integrating AI into diagnostic tools such as radiology software that can detect cancerous lesions with high precision and predictive models that help assess treatment responses. Advanced machine learning models facilitate real-time analysis of complex medical data, assisting oncologists in making informed decisions with speed (10). AI models like convolutional neural networks (CNNs) are being used to enhance the accuracy and speed of imaging analysis of modalities such as MRI, CT, and PET scans, detecting abnormalities with precision comparable to, or even exceeding that of experienced radiologists (11). In terms of prognosis, AI helps predict disease outcomes and patient survival by analysing large-scale datasets from clinical trials and patient records. This capability assists in risk stratification and treatment decisions, ultimately improving patient outcomes (9). AI hastens drug discovery by identifying promising drug candidates and predicting their interactions at a molecular level, making cancer treatment research more efficient (1).

**Figure 1:**
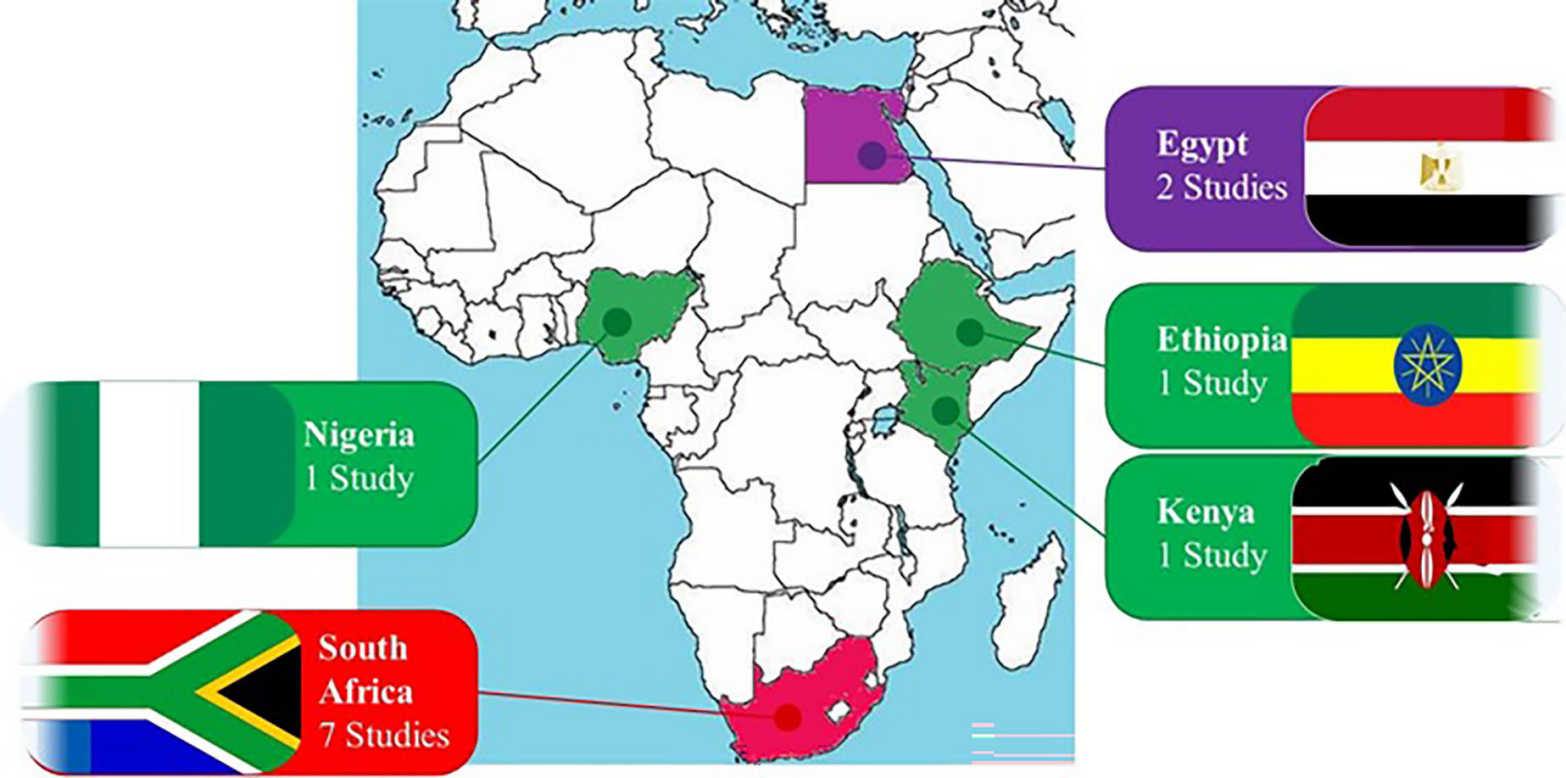
Oncological AI-based prediction models in Africa by regions. The deployment and effectiveness of AI-based oncological prediction models in Africa vary significantly across different regions, reflecting diverse socio-economic, infrastructural, and healthcare contexts.

In Africa, the potential for AI to impact oncology is substantial, given the continent’s unique healthcare landscape (12). The Type II EC burden in Africa is marked by late-stage diagnosis, limited access to specialized care, and significant resource constraints (13). AI technologies present an opportunity to bridge gaps in diagnostic accuracy and access to expert care, even in remote areas (5), affording patients in rural areas the opportunity to receive specialist consultation without travelling to urban centre’s (7). AI-powered mobile apps can support awareness campaigns and self-screening initiatives, educating the population about early warning signs and encouraging timely medical consultations (5). AI tools can assist in the training of healthcare workers by providing decision-support systems that enhance their diagnostic skills and treatment planning capabilities (13). Despite these advances, challenges such as data privacy, the need for large, annotated datasets, and the complexity of integrating AI with existing healthcare systems remain (12). While AI holds significant potential for transforming healthcare in Africa. In contrast to the global north, the under-developed technological/digital infrastructure, unstandardized data collection and storage protocols, limited healthcare resources, unregulated frameworks that govern the use of AI in healthcare and the shortage of healthcare professionals with AI expertise in Africa, all limit the adoption and effective use of these technologies (2). The deployment and effectiveness of AI-based oncological prediction models in Africa vary significantly across different regions, reflecting diverse socio-economic, infrastructural, and healthcare contexts as shown in **Figure 1** (12).

Tailored solutions that consider the unique healthcare landscape of Africa are essential to bridge the gap between AI advancements in the developed countries and those in Africa (2). While the continent faces the aforementioned barriers, there is also a growing realization towards developing context-specific AI-powered therapeutic solutions which are crucial in dealing with the rising incidence of Type II EC (13). Establishing robust frameworks to manage patient data, ensure algorithm transparency, and align AI use with global ethical standards is critical to the development of practice changing AI-directed strategies on the continent (12). Once these challenges are addressed through targeted efforts, coupled with adaptive technologies and collaborative strategies to harness the power of AI, the potential for positive impact across the African continent is immense (13). This review explores the global trends in AI applications for Type II EC and its potential to transform oncology care in Africa into a more precise, personalized and efficient field The article provides a comprehensive overview of how AI can be leveraged to meet the continent’s unique healthcare needs, reduce healthcare disparities and improve type II EC patient outcomes in Africa.

## 2 The epidemiology of type II EC in Africa

The epidemiology of Type II EC in Africa is influenced by a complex interplay of demographic, socioeconomic, genetic, and healthcare system factors (13). In the Type II EC group, serous and clear cell histologies are the most commonly occuring subtypes, and they are significantly more aggressive and less prevalent than Type I EC (3). In general, EC is less common in sub-Saharan Africa compared to more developed regions, where it is the most frequently diagnosed gynecologic malignancy (14). In developed countries, despite Type I EC being the most prevalent subtype, the incidence of Type II EC is on the rise, and this is attributed to longer lifespans, as these poorly differentiated cancers are more prevalent in the elderly (7). Additionally, the global obesity epidemic is thought to indirectly influence these trends, despite Type II EC having less of an association with obesity compared to Type I EC (3). The mortality rates from Type II EC are disproportionately high relative to its incidence (15). This high mortality is attributed to their aggressive phenotype, as characterised by their rapid progression and a high propensity to metastasize (7).

The incidence of Type II EC in Africa may be underestimated due to limited cancer registries and underreporting. Type II EC, while representing a smaller proportion of all EC cases, accounts for most of the EC-related mortality as it’s often diagnosed at an advanced stage (13). The peak incidence for Type II EC is in older populations, which aligns with demographic shifts and increased life expectancy in parts of Africa (3). According to Sponholtz and coworkers, women of African descent are at a higher risk of developing Type II EC as compared to white women (16, 17). A history of tamoxifen use, prior pelvic radiation, and genetic syndromes like Lynch syndrome may also increase the risk (18).

Unlike Type I, which is often hormone-dependent and has a better prognosis, Type II EC typically occurs in postmenopausal women and is associated with a higher rate of metastasis at diagnosis (2, 3). The risk factors for Type II EC differ from those of Type I. With obesity, unopposed estrogen exposure, and metabolic syndrome being major risk factors for Type I, while Type II EC is less associated with hormonal factors as it’s more prevalent in postmenopausal women, occurs at a higher rate in women of African descent, and is associated with high risk genetic mutations, such as TP53 (7). The increased risk in African females, is thought to have a ggenetic basis as alterations in genes like TP53 and PTEN are commonly found in Type II EC. Additionally, genetic syndromes like Lynch Syndrome, which is characterized by mutations in mismatch repair genes, also increase the risk of EC. Research on genetic variants among African populations may shed light on specific susceptibilities and help in developing targeted therapies and personalized treatment approaches. Understanding these predispositions is crucial for improving early detection, diagnosis and treatment outcomes for EC in African women (15).

Limited healthcare access, inequalities and delayed diagnosis contribute to the higher morbidity and mortality associated with Type II EC in African regions (13). Many African countries lack widespread screening programs for early detection of gynecological cancers. As a result, Type II EC is often diagnosed at an advanced stage when treatment options are less effective (19) The treatment options typically involve a combination of surgery, radiation, and chemotherapy, but the prognosis becomes guarded when the disease has spread beyond the uterus, making potentially curative localised surgical approaches less feasible (3, 15). The lack of specialized oncological services and trained healthcare professionals exacerbates treatment challenges for aggressive cancers like Type II EC (8, 13). Delayed health-seeking behaviours by patients, either because of misconceptions about cancer, low levels of public health awareness and fears of Stigmatisation may affect early diagnosis rates and negatively affect patient outcomes (19).

Type II EC is notorious for its resistance to standard chemotherapy treatment protocols, which is often the only treatment option available, as many patients’ are diagnosed with metastatic disease at diagnosis. Making surgery and/or radiation non-viable therapeutic options (3, 17). Targeted therapies are still in development globally, however, their high cost would make them unaffordable to many African countries, limiting their access and use on the continent. The standard of care in many African regions still heavily relies on conventional chemotherapy regimens, with limited use of personalized medicine and their companion molecular diagnostics (13). There are limited published studies specifically focusing on the comprehensive epidemiology of Type II EC in Africa, making it difficult to draw definitive conclusions (15, 19). However, the existing data suggests that the disease is under-recognized and underreported, as many African countries have low diagnostic capacity for malignancies and lack formal cancer registries to adequately document diagnosed cases (19). The burden may increase as lifestyles change, leading to higher obesity rates and longer life expectancies, which are known risk factors for EC (13).

Based on their aggressive nature, i.e. accounting for most of the EC-related mortality and higher likelihood of late stage presentation. Early diagnosis of Type II EC is crucial in obtaining control of the disease (7). Unlike Type I EC, which often presents with symptoms like abnormal uterine bleeding, Type II cancers are more likely to be asymptomatic until they reach an advanced stage (15). When detected early, Type II ECs can be treated more effectively with localised approaches, such as surgery and/or radiation, improving overall survival rates and reducing the likelihood of metastasis (3, 7). With studies having shown that when Type II EC is diagnosed and treated at an early stage, the 5-year survival rate can be significantly higher compared to those diagnosed at a later stage (14). However, early diagnosis remains challenging due to the lack of specific symptoms with low stage disease (7). Routine screening for EC is not currently recommended for asymptomatic women, which means that Type II EC are often only discovered incidentally or at an advanced stage (15).

Given the heterogeneity of Type II ECs, personalized treatment approaches are essential (2). Personalized medicine involves tailoring treatment based on the genetic, molecular, and clinical characteristics of the tumor, offering a more targeted and effective approach compared to traditional therapies v (17). Recent advances in genomic and molecular profiling have identified distinct genetic mutations and pathways involved in Type II EC, such as p53 mutations, HER2 overexpression, and alterations in the PI3K/AKT/mTOR pathway (9). These discoveries have opened new avenues for targeted therapies, which are more specific and generally have a more tolerable side effect profile than conventional chemotherapy (7). The development of targeted therapies, such as HER2 inhibitors and PI3K/AKT/mTOR pathway inhibitors, has shown promise in treating Type II ECs. AI plays a significant role in analyzing large genetic datasets, identifying alterations or overexpressed proteins, while predicting which patients are likely to benefit from targeted therapies that inhibit these pathways. These therapies are designed to target specific molecular alterations in cancer cells, leading to more precise and effective treatment outcomes (3). Immunotherapy is another area of personalized treatment that is gaining traction in the management of Type II EC (7). By harnessing the patient’s immune system to target cancer cells, immunotherapy offers a novel treatment modality, particularly for tumors that are resistant to conventional chemotherapy (13).

Personalized care not only improves the efficacy of the therapy being used, but also minimizes the risk of toxic bystander adverse effects, which in turn enhances the patients quality of life (3). By focusing on the specific characteristics of each tumor, personalized medicine has the potential to improve treatment outcomes significantly, particularly in cases of recurrent or metastatic Type II EC (2). The importance of early diagnosis and personalized treatment approaches in Type II EC cannot be overstated. As our understanding of the molecular underpinnings of Type II EC continues to grow, so too will the opportunities to develop more targeted and personalized therapeutic strategies, ultimately improving outcomes for patients with this aggressive form of cancer (9). Thus, the epidemiology of Type II EC in Africa underscores significant challenges related to diagnosis, treatment, and access to healthcare. While the disease represents a smaller subset of EC cases, its disproportionate impact on mortality rates necessitates focused attention and research (13). Addressing these gaps through improved healthcare access, enhanced screening, and targeted research could lead to better outcomes for women affected by this aggressive cancer type (13, 19).

## 3 The role of artificial intelligence in oncology

Artificial Intelligence (AI) has emerged as a revolutionary tool in the field of oncology, promising to enhance various aspects of cancer care, from early diagnosis to personalized treatment planning (9). AI encompasses a range of technologies, including machine learning (ML), deep learning (DL), and other AI-based approaches that enable computers to learn from data, identify patterns, and make decisions with minimal human intervention (13, 19). AI in oncology primarily leverages machine learning and deep learning. Machine learning involves training algorithms on large datasets to identify patterns and make predictions (20). Deep learning, a subset of machine learning, employs artificial neural networks to model complex relationships in data, making it particularly effective for interpreting medical images, genomic data, and other complex datasets (2). Other AI technologies, such as natural language processing (NLP), play a crucial role in extracting valuable information from unstructured data sources, including electronic health records and scientific literature by enabling AI systems to interpret human language and identify clinically relevant patterns (9).

AI has shown significant potential in cancer diagnosis by enhancing the accuracy and speed of imaging analysis (2). For instance, deep learning algorithms can analyze mammograms, CT scans, and MRIs to detect abnormalities with a level of precision comparable to, or even exceeding, that of experienced radiologists (13). AI is also used in pathology, where it aids in the analysis of histopathological images to identify cancerous tissues, thus reducing human error and improving diagnostic consistency (19). In treatment planning, AI tools assist oncologists in personalizing therapy by integrating and analyzing complex datasets, including genomic profiles, patient histories, and clinical trial data (20). AI models can predict which therapies will be most effective for individual patients based on the molecular characteristics of their tumors (9). Furthermore, AI algorithms can optimize radiation therapy planning by accurately segmenting tumors and critical structures, thereby minimizing exposure to healthy tissues (3). AI also has significant applications in prognosis, where it helps predict disease outcomes and patient survival (7). By analyzing large-scale datasets from clinical trials and patient records, AI models can identify patterns that correlate with specific prognostic outcomes, assisting in risk stratification and treatment decisions (2).

The transformative potential of AI in oncology lies in its ability to handle vast amounts of complex data, uncover hidden patterns, and provide insights that may not be apparent to human clinicians (13). This capability can lead to earlier detection of cancer, more accurate diagnoses, and personalized treatments, ultimately improving patient outcomes (19). AI also has the potential to democratize access to high-quality cancer care, particularly in low-resource settings, by enabling remote diagnosis and treatment planning through telemedicine platforms (7). Moreover, AI-driven predictive models can accelerate drug discovery by identifying promising therapeutic targets and optimizing clinical trial design, thereby reducing the time and cost associated with bringing new cancer therapies to market (20). As AI technologies continue to advance, they hold the promise of transforming oncology into a more precise, personalized, and efficient field, offering new hope for patients worldwide (13). In the African context, refining cancer-focused AI platforms offers potential to improve outcomes in diseases such as Type II EC (21). However, adoption has been comparatively slow, due in part to the need for context-specific validation of AI tools within local clinical environments. Additional barriers include the heterogeneity of cancer subtypes at molecular and etiological levels, as well as regional differences such as the quality of healthcare infrastructure and cultural approaches to cancer care (21). **Table 2** illustrates the stages of application and implementation of oncological AI tools across Africa.

**Table 2:** Phases of oncological AI tool application and implementation in Africa.

Year	AI tool	Predicted outcome(s)	Phases	Reference
2018	Machine learning	Breast cancer staging	Phase I, II, IV	Hamouda et al., 2018
2020	Deep learning	Contouring of clinical treatment volumes and normal structures in cervical cancer radiotherapy	Phase III	Rhee et al., 2020
2020	Natural language processing	Identification of malignant cases in electronic records	Phase I-III	Achilonu et al., 2021
2021	Machine learning	Cell-free-DNA-based oesophageal cancer diagnosis	Phase I-III only	Kandimalla et al., 2021
2021	Machine learning	Post-operative length of stay after colorectal cancer (CRC) resection	Phase I-III	Achilonu et al., 2021a
2021	Deep learning	CRC recurrence and survival	Phase I-III	Achilonu et al., 2021b
2021	Machine learning	Breast cancer tumor mutation burden	Phase I-III	Nassar et al., 2021
2021	Machine learning	Breast cancer risk prediction	Phase I-III	Macaulay et al., 2021
2021	Natural language processing	Identification of malignant cases in electronic records	Phase I-III	Olago et al., 2020
2021	Deep learning	Detection of HSIL and LSIL in cervical cance	Phase I-III	Holmström et al., 2021
2021	Machine learning	Detection and typing of leukemic cells	Phase I-IV	Dese et al., 2021
2022	Natural language processing	Identification of demographic, clinical and molecular subtype information from records	Phase I-III	Achilonu et al., 2022

**Figure 2:**
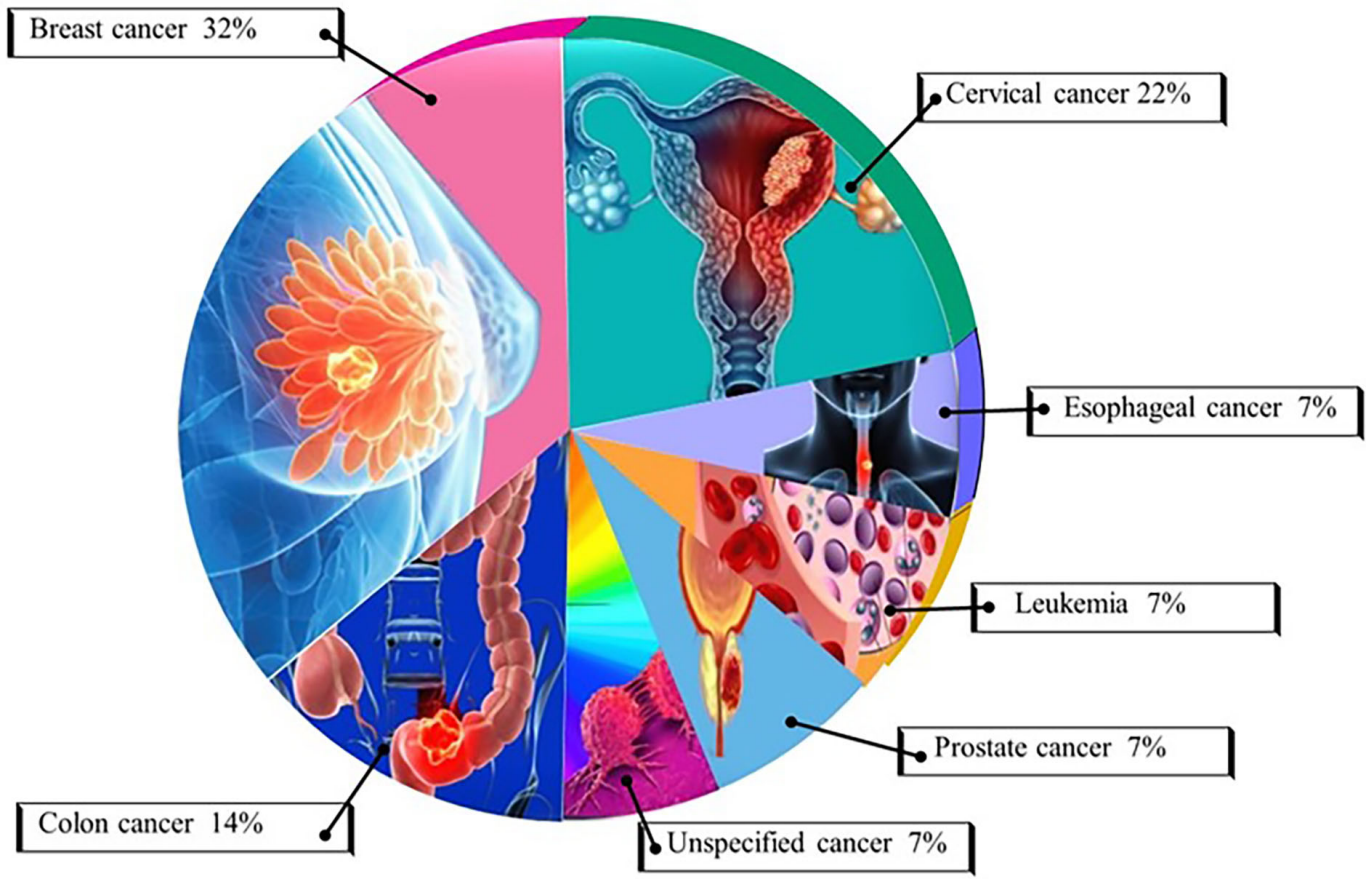
African oncological AI-based prediction models by cancer type. AI-driven prediction models for oncology in Africa are gradually expanding, with a primary focus on breast, cervical, colorectal, and prostate cancers. These efforts largely mirror the regional cancer burden, with more developed economies leading to AI development. Further advancements in AI technology for cancer in Africa will depend on improved data collection, validation studies, and infrastructure expansion to enhance accessibility and outcomes in underserved regions.

Generally, AI models for cancer care in the region are relatively recent, with notable reports emerging mainly since 2018 (21). Reflecting economic and infrastructure diversity, the uptake of AI tools varies across African subregions, with development efforts concentrated in nations like South Africa, Egypt, Nigeria, and Kenya (as shown in **Figure 1**), where technological capabilities and resources are more advanced (22). However, this leaves approximately 90% of African countries, particularly in Central Africa and other low-income or lower-middle-income regions, with limited AI deployment for cancer care (23). Oncological AI applications in Africa have focused predominantly on improving outcomes for breast, cervical, and colorectal cancers (**Figure 2**), which align closely with the continent’s most prevalent cancers. Several innovative AI tools are being developed to address unique African healthcare challenges, with AI-powered diagnostic tools, remote diagnostics and mobile Apps, personalized treatment planning, training and decision-support systems making significant strides in improving diagnostic accuracy, expanding access to expert care and supporting personalised oncological care (21).

However, despite several AI tools being used in the global north that continue to make oncology more precise, personalized and efficient. There are currently limited AI-driven platforms under development or in use for managing Type II EC in Africa. Although EC models have been suggested to primarily support incidence reporting through cancer registry data from pathology reports, Type II EC models would best impact on diagnostic or treatment processes (24). There is a significant need for both the development of new AI platforms and the external validation of existing ones to address ECs, particularly the less common Type II subtypes (25). Type II EC, which has higher age-standardized incidence rates in Africa than in other regions, would benefit substantially from AI-powered predictive tools (12). The advancements could significantly elevate the standards of diagnosis and treatment in Africa, improving the overall quality of cancer care across the continent, reducing healthcare disparities and offering new hope to patients (21).

## 4 Current state of AI in type II EC research

**Figure 3:**
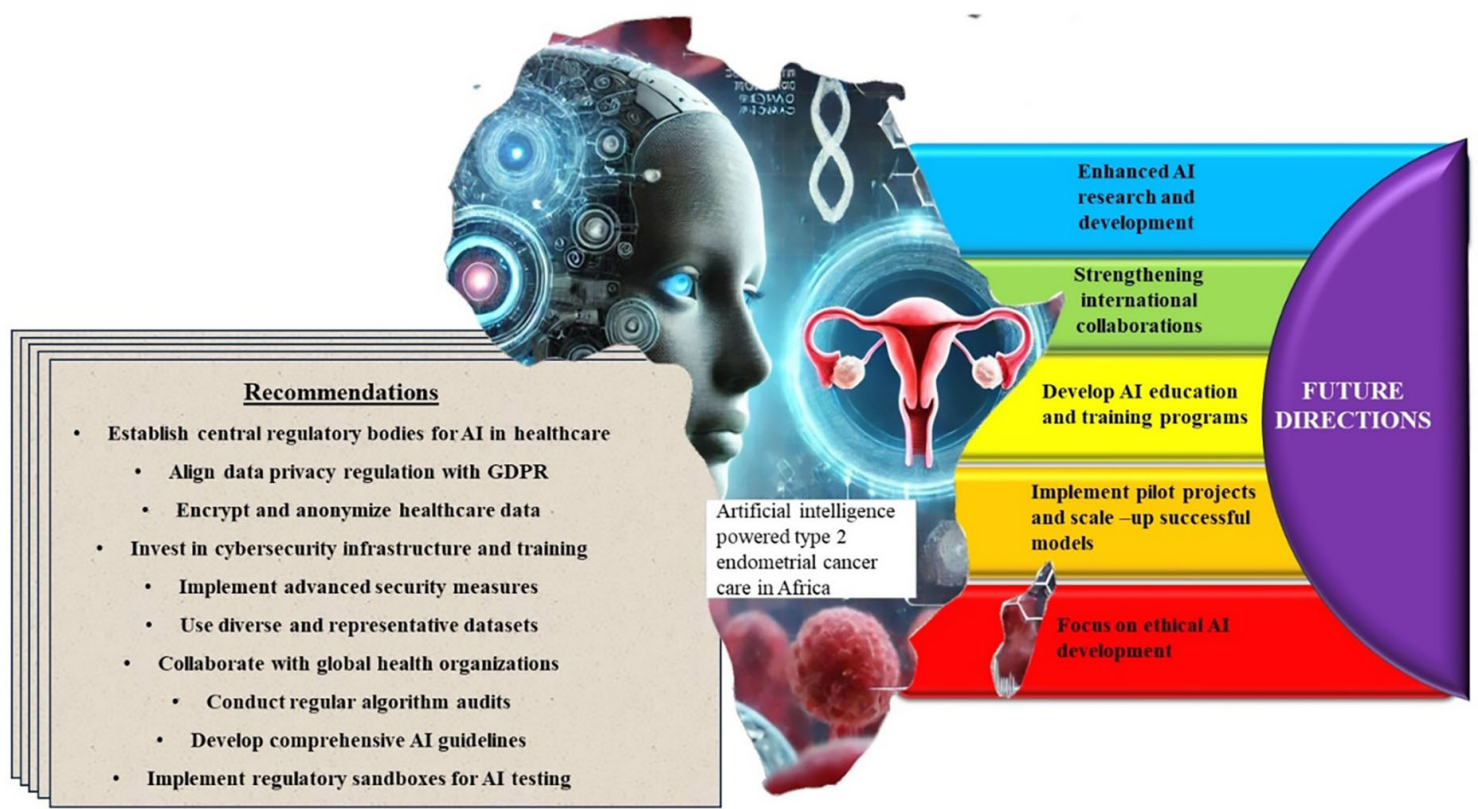
Oncological AI-based prediction models in Africa modality of application. AI-based oncological prediction models hold immense potential for improving cancer outcomes in Africa. AI tools are used in pilot projects to boost the efficiency of screening programs for common cancers, such as breast and cervical cancer. Large datasets from hospital records and clinical trials are processed using AI to identify trends and insights that can inform policy and treatment approaches. AI algorithms are integrated into pathology labs to aid in faster and more accurate analysis of biopsy samples.

AI is revolutionizing the field of histopathology by enabling more accurate and efficient analysis of tissue samples (26). As shown in **Figure 3**, AI-based oncological prediction models hold immense potential for improving cancer outcomes in Africa. AI tools are used in pilot projects to boost the efficiency of screening programs for common cancers, such as breast and cervical cancer (12). Large datasets from hospital records and clinical trials are processed using AI to identify trends and insights that can inform policy and treatment approaches. AI algorithms are integrated into pathology labs to aid in faster and more accurate analysis of biopsy samples (13). ML algorithms, particularly deep learning (DL) models such as convolutional neural networks (CNNs), have shown strong performance in analyzing histopathological images of endometrial biopsies. CNNs are specifically designed to detect spatial features in visual data, enabling accurate identification and classification of cancerous cells (27). These AI tools can differentiate between Type I and Type II ECs by recognizing subtle histological patterns that may be missed by human pathologists (3). Studies have demonstrated that AI-based image analysis can significantly improve diagnostic accuracy, reduce interobserver variability, and speed up the diagnostic process (9). AI has shown potential in identifying novel biomarkers for the early detection of Type II EC. By analyzing large datasets of genomic, transcriptomic, and proteomic information, AI algorithms can discover patterns and correlations that indicate the presence of early-stage cancer (19). These AI-driven biomarkers can be used to develop non-invasive screening tests, potentially leading to earlier diagnosis and better outcomes for patients. Recent research has focused on using AI to integrate multi-omics data to enhance the sensitivity and specificity of these biomarkers (15).

The performance of these AI models is typically evaluated using metrics such as sensitivity, specificity, accuracy, and the Area Under the Receiver Operating Characteristic Curve (AUC), which provide insight into their clinical reliability and predictive strength.

AI is being integrated with imaging modalities like MRI and CT scans to improve the accuracy of Type II EC diagnosis (20). AI algorithms can analyze complex imaging data to identify tumors, assess their size and extent, and differentiate between benign and malignant lesions (13). The integration can help in better staging of the cancer, which is crucial for treatment planning. Additionally, AI-powered imaging can enhance the detection of metastasis, improving the overall diagnostic process (7). AI is playing a pivotal role in the development of personalized treatment plans for Type II EC. By analyzing patient data, including genetic profiles, tumor characteristics, and response to previous treatments, AI models can recommend personalized therapeutic strategies (15). These models consider various factors, such as the likelihood of treatment success, potential side effects, and patient preferences, to optimize treatment outcomes (20). The approach is particularly beneficial for patients with Type II EC, which is often more aggressive and has a poorer prognosis compared to Type I cancer (2).

AI is also being used to predict how patients with Type II EC will respond to specific treatments (20). Machine learning models can analyze data from previous clinical trials and real-world patient outcomes to predict the effectiveness of chemotherapy, radiation therapy, and immunotherapy (13). These predictions can help oncologists choose the most appropriate treatment for each patient, reducing the risk of ineffective therapy and improving overall survival rates (15). Risk stratification is essential in managing Type II EC, and AI tools are proving to be invaluable in this area (2). AI models can analyze a wide range of patient data, including genetic information, tumor characteristics, and lifestyle factors, to classify patients into different risk categories (27). The stratification helps in tailoring treatment strategies to the specific needs of each patient and can also identify those who may benefit from more aggressive treatment or closer monitoring (9). AI-based prognostic models are being developed to predict the likelihood of cancer recurrence and overall survival in patients with Type II EC (27).

These models use a combination of clinical, pathological, and molecular data to generate individualized predictions (2). Such tools are particularly valuable in guiding post-treatment surveillance and in making informed decisions about adjuvant therapy (9). The use of AI in prognosis is expected to lead to more accurate predictions and better-informed clinical decisions (27). Recent studies have highlighted the significant impact of AI on various aspects of Type II EC management (2). For example, research has demonstrated the utility of AI in enhancing the accuracy of histopathological diagnoses, identifying novel biomarkers for early detection, and developing personalized treatment plans (15). Additionally, studies have shown that AI-driven prognostic models can improve the prediction of recurrence and survival, leading to better patient outcomes (13). These advances underscore the potential of AI to transform the diagnosis, treatment, and management of Type II EC, ultimately improving patient care (2).

## 5 Challenges and opportunities for AI in type II EC care in Africa

The technological infrastructure in Africa for supporting AI in healthcare is still in its nascent stages, with significant disparities across the continent (2). While some urban centers in countries like South Africa, Kenya, and Nigeria have started to integrate AI into healthcare, many regions lack the necessary infrastructure, such as high-speed internet, advanced computing power, and robust data storage facilities (13). The uneven distribution of technology across the continent limits the widespread adoption of AI in healthcare, including in the diagnosis and treatment of Type II EC (19). In low-resource settings, several barriers hinder the implementation of AI technologies. These include limited access to advanced diagnostic tools, inadequate healthcare funding, and a shortage of trained professionals who can operate AI systems (15). Additionally, the high cost of AI technology and its maintenance poses a challenge for healthcare facilities with constrained budgets (2). These barriers result in a digital divide, where only a few well-funded institutions can access and benefit from AI, while the majority of healthcare providers in rural and underserved areas are left behind (13). Yet, realizing the full potential of these innovations requires addressing foundational limitations in data infrastructure and representation.

A significant challenge in applying AI to Type II EC care in Africa is the scarcity of high-quality, large-scale datasets (13). Many African healthcare facilities lack the digital infrastructure needed to collect, store, and manage vast amounts of patient data (19). Additionally, existing data are often fragmented, incomplete, or inconsistent, making it difficult to train robust AI models (27). The lack of standardized data collection protocols further exacerbates this issue, hindering the development of AI systems that require comprehensive and accurate datasets (7). For AI models to be effective in Africa, it is crucial that the datasets used for training these models are representative of the diverse populations across the continent (13). However, most AI models are trained on data from high-income countries, which may not reflect the genetic, environmental, and socioeconomic factors prevalent in African populations (9). This lack of diversity in training data can lead to biases in AI algorithms, resulting in inaccurate diagnoses and treatment recommendations for African patients (13). Ensuring that AI models are trained on diverse and representative datasets is essential for their successful application in African healthcare (7).

AI models trained on non-representative datasets can perpetuate biases, leading to disparities in healthcare outcomes (19). For example, AI systems developed based on data from Western populations may not accurately predict disease risk or treatment responses in African populations (13). This can result in misdiagnoses or inappropriate treatment plans, exacerbating existing healthcare inequalities. Addressing these biases is critical to ensuring that AI technologies benefit all populations, including those in Africa. The use of AI in healthcare raises several ethical concerns, particularly in the context of Africa (13). Issues such as the potential for algorithmic bias, the lack of transparency in AI decision-making processes, and the risk of exacerbating existing inequalities are of significant concern (28). There is also the ethical dilemma of relying on AI for critical healthcare decisions in settings where there may be a lack of human oversight or the ability to challenge AI recommendations (29). Ensuring that AI is used ethically and equitably in African healthcare requires careful consideration of these issues (20).

The development of regulatory frameworks and guidelines for AI in healthcare is still in its early stages in most African countries (13). While some nations are beginning to formulate policies around AI, there is a general lack of comprehensive legal frameworks to govern their use in healthcare (28). The absence of regulation raises concerns about the quality and safety of AI applications, particularly in sensitive areas like cancer diagnosis and treatment (15). Establishing clear regulatory guidelines is essential to ensure that AI technologies are deployed safely and responsibly (20). The use of AI in healthcare involves the collection and analysis of large amounts of sensitive patient data, raising concerns about privacy, data security, and patient consent (13). In Africa, where data protection laws may be less stringent or poorly enforced, there is a risk that patient data could be misused or inadequately protected (20). Ensuring that patients are fully informed about how their data will be used and obtaining their consent is crucial (2). Additionally, robust data security measures must be in place to protect patient information from breaches or unauthorized access (29).

The successful integration of AI into Type II EC care in Africa requires a workforce that is knowledgeable about AI technologies (19). Currently, there is a shortage of healthcare professionals with expertise in AI, which limits the adoption and effective use of these technologies (13). Building capacity in AI among healthcare providers is essential to ensure that they can leverage AI tools to improve patient care. This includes not only technical skills but also an understanding of the ethical, legal, and practical implications of AI in healthcare (2). To address the skills gap, there is a need for targeted training programs that enhance AI literacy among healthcare professionals in Africa. These programs can be developed in collaboration with academic institutions, industry partners, and international organizations (19). Training initiatives should focus on both the technical aspects of AI, such as data analysis and machine learning, as well as the practical application of AI in clinical settings (13). Additionally, fostering collaborations between African institutions and global AI experts can facilitate knowledge transfer and capacity building (29). Academic institutions and research organizations in Africa have a critical role to play in advancing AI in healthcare (19). These institutions can lead research on AI applications in Type II EC, develop context-specific AI tools, and contribute to the global discourse on AI ethics and regulation (13). Moreover, they can serve as hubs for training the next generation of AI-savvy healthcare professionals (19). By fostering interdisciplinary research and collaboration, academic institutions can help bridge the gap between AI research and its practical application in African healthcare (29).

## 6 African innovations and contributions in AI for type II EC

African-led AI initiatives and projects in healthcare have gained significant momentum in recent years, with the aim of improving cancer care on the continent, including that of Type II EC (13). For example, the African Institute for Mathematical Sciences (AIMS) has been instrumental in fostering AI research across the continent, with a focus on addressing local healthcare challenges (13). Okolo and his coworkers have described how researchers at AIMS have developed AI models that can analyze large datasets for early cancer detection, improving diagnostic accuracy in resource-limited settings (26). Another of such initiatives is the University of Cape Town’s Data Science for Social Impact Research Group, which has applied machine learning techniques to analyze cancer data and predict patient outcomes (30). These initiatives demonstrate the growing capacity within Africa to lead AI research that is not only relevant to the continent’s unique healthcare needs but also contributes to the global AI landscape (13). However, while artificial intelligence holds great promise, its integration into African healthcare systems faces critical challenges. Most AI models are trained on datasets derived from high-income countries, which often do not reflect the demographic, genetic, or clinical diversity of African populations. This can lead to model bias, reduced diagnostic accuracy, and disparities in healthcare outcomes when applied in low-resource settings. Additionally, the limited availability of high-quality local data—combined with infrastructural, computational, and annotation constraints—hampers the generalizability and real-world effectiveness of these tools within African contexts. The impact of these efforts is evident in improved diagnostic tools, better patient outcomes, and the increased visibility of African researchers in the global AI community (30).

Collaboration between African institutions and global partners has also been key to advancing AI research in healthcare (19). The AI4D Africa program, supported by the International Development Research Centre (IDRC) and the Swedish International Development Cooperation Agency (Sida), has funded numerous projects that leverage AI to address health challenges, including cancer (30). One such project is the collaboration between the University of Lagos and the Massachusetts Institute of Technology (MIT), which focuses on developing AI-driven diagnostic tools for cancer detection (30). These collaborations not only provide African researchers with access to advanced technology and expertise but also ensure that the AI solutions developed are relevant to the African context (26). By working together, African institutions and their global partners are driving innovation in AI for cancer care and ensuring that these innovations are accessible to those who need them most (30).

The development of AI tools tailored to Africa’s unique healthcare challenges is crucial for their success (29). For Type II EC, this means creating AI models that account for the specific genetic, environmental, and socioeconomic factors prevalent in African populations (2). For instance, AI-driven tools that analyze histopathological images have been adapted to better suit the characteristics of cancer presentations in African women (30). These tools assist pathologists in making accurate diagnoses, despite working in regions that lack advanced diagnostic resources (15). Additionally, African developers are creating mobile-based AI applications to improve accessibility in remote areas (20). These apps allow healthcare workers to input patient data and receive AI-generated recommendations for further diagnostic testing or treatment, thus bridging the gap in healthcare delivery in underserved regions (26).

Several locally developed AI technologies are making significant contributions to cancer care in Africa (20). In Kenya, a team of researchers from Strathmore University developed an AI-based tool to assist in the early detection of cervical cancer. This tool uses machine learning algorithms to analyze medical images and identify early signs of cancer, thereby enabling timely intervention (30). Adebamowo et al. showed that the Health AI Initiative in Nigeria has developed AI-powered software that assists oncologists in planning treatment protocols for cancer patients, including those with Type II EC (2). The software is designed to work with the specific healthcare infrastructure available in Nigerian hospitals, making it a valuable tool for improving cancer care in the country (30). These initiatives demonstrate the growing capacity within Africa to lead AI research that is not only relevant to the continent’s unique healthcare needs but also contribute to the global AI landscape and the visibility of African researchers in the global AI community.

Incorporating indigenous knowledge into AI-driven healthcare represents a unique opportunity for innovation in Africa (20). Indigenous knowledge systems, which include traditional medical practices and an understanding of local disease patterns, can provide valuable insights that enhance the effectiveness of AI models (7). For example, AI systems can be trained using data that includes information on traditional remedies and local health practices, allowing these systems to offer recommendations that are culturally appropriate and more likely to be accepted by local populations (9). Furthermore, leveraging indigenous knowledge in AI development can lead to the discovery of novel biomarkers or treatment approaches that are specific to African populations (13). By integrating this knowledge, AI tools can offer more personalized and effective healthcare solutions for patients with Type II EC (7).

Africa’s rich genetic diversity presents a unique opportunity for AI-driven research. Genomic studies conducted in African populations have the potential to uncover novel genetic markers associated with Type II EC, which may not be present in other populations (20). AI tools can analyze these genomic datasets to identify patterns and correlations that could lead to new diagnostic methods or targeted therapies (3).Moreover, epidemiological data from Africa, which reflects the continent’s unique environmental and lifestyle factors, can be used to train AI models that predict cancer risk and treatment outcomes more accurately for African patients (30). This approach not only enhances the relevance of AI applications in Africa but also contributes to the global understanding of cancer biology and its treatment (26).

## 7 The future of AI in type II EC care in Africa

The future of AI in Type II EC care is marked by several emerging trends and innovations that promise to revolutionize diagnostics, treatment planning, and patient outcomes in Africa (3). AI technologies such as deep learning and neural networks are becoming increasingly sophisticated, enabling the development of algorithms that can analyze complex medical data with unprecedented accuracy (4, 7). These technologies are particularly relevant for histopathological image analysis, where AI can assist in identifying cancerous cells with greater precision than traditional methods (2). Moreover, the integration of AI with imaging modalities like MRI and CT scans is advancing. AI-driven tools are being developed to enhance the resolution and interpretability of these images, facilitating earlier and more accurate detection of Type II EC (31). These advancements are critical in resource-limited settings where access to highly trained specialists may be scarce.

AI research in Africa is poised to focus on creating context-specific solutions that address the unique challenges of the continent (30). This includes developing AI models that are trained on local datasets, ensuring that the algorithms are tailored to the genetic and environmental factors prevalent in African populations (13). There is also a growing interest in using AI to facilitate telemedicine and mobile health (mHealth) initiatives, which could dramatically improve access to cancer care in remote and underserved areas (9). In addition, collaborations between African research institutions and international partners are likely to increase, driving innovation and the development of AI tools that can be deployed in both African and global healthcare systems (19). The future of AI in Africa will also involve the integration of AI with other emerging technologies, such as genomics and bioinformatics, to create more personalized and effective treatment plans for patients with Type II EC (20).

**Figure 4:**
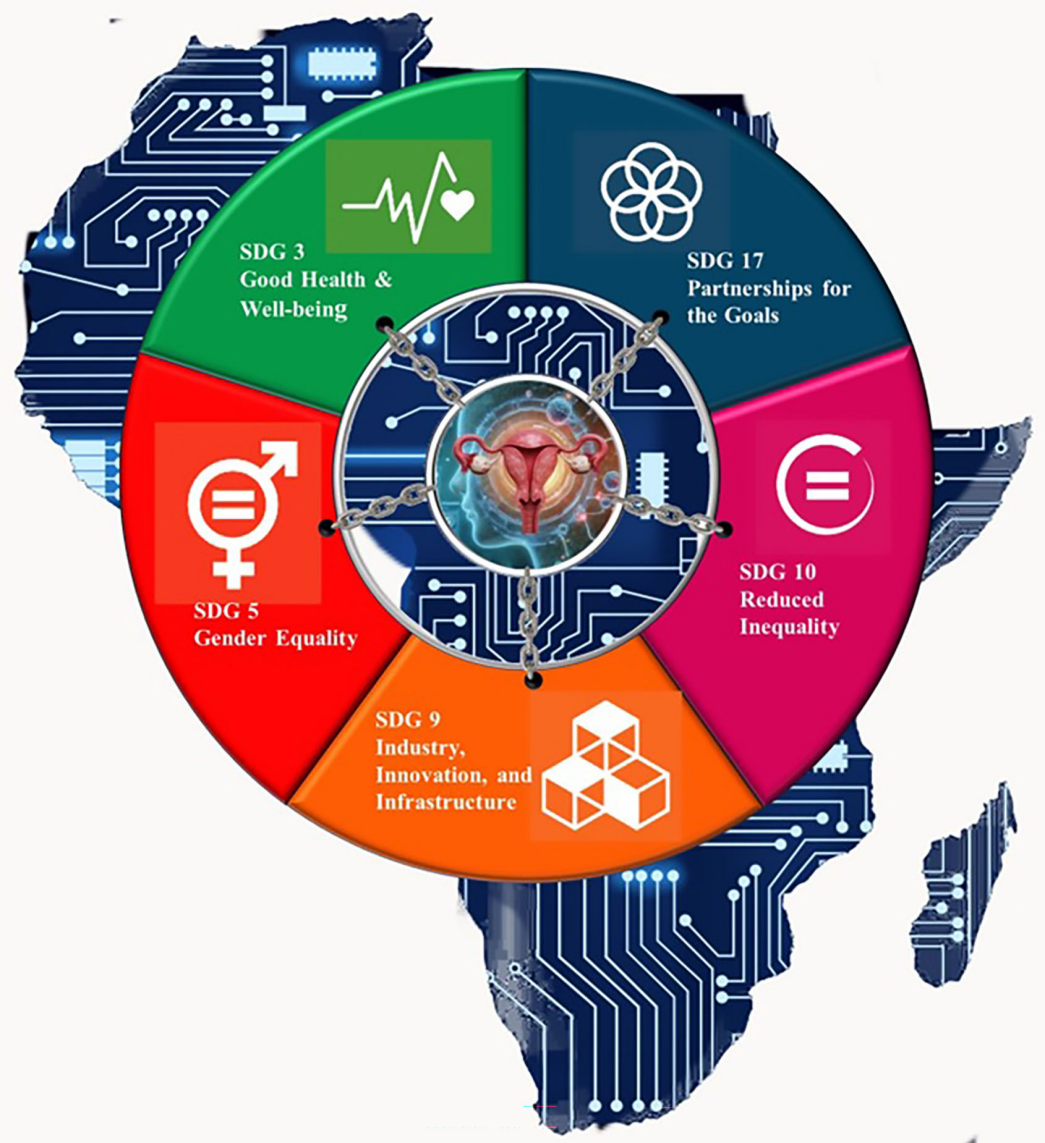
AI & Type II EC Care in Africa: Recommendations and Future Directions. To address challenges unique to the continent several key recommendations have been proposed which include establishing robust, region-specific cancer registries and datasets is essential for training and validating AI models, fostering collaborations between African research institutions and global AI experts can help tailor advanced tools to local contexts and enhancing accessibility through cost-effective, scalable AI solutions that work in low-resource settings is crucial. Future directions should focus on leveraging AI for underserved malignancies like Type II EC while aligning innovations with global health frameworks, such as the Sustainable Development Goals (SDGs), to promote equitable and effective Type II EC management across the continent.

The implementation of AI in Type II EC care has the potential to significantly improve healthcare outcomes in Africa as shown in **Figure 4** (26). AI can enhance diagnostic accuracy by analyzing medical images and other patient data to detect cancer at earlier stages, which is crucial for improving survival rates (20). For instance, AI algorithms can identify subtle patterns in histopathological images that may be missed by human eyes, leading to earlier and more accurate diagnoses (3). In terms of treatment, AI can help in developing personalized treatment plans that take into account the genetic makeup of the patient, the characteristics of the tumor, and the patient’s overall health (29). This personalized approach can increase the effectiveness of treatments, reduce side effects, and improve the quality of life for patients (2). AI can also be used to predict treatment responses and outcomes, allowing clinicians to make more informed decisions about the best course of action for each patient (27).

One of the most significant benefits of AI in healthcare is its potential to reduce disparities in access to care (26). In many parts of Africa, healthcare resources are limited, and access to specialist care is often restricted to urban areas (15). AI-driven solutions, such as mobile diagnostic tools and telemedicine platforms, can help bridge this gap by providing remote consultations and diagnostic services to patients in rural and underserved areas (2). Additionally, AI can help optimize the allocation of resources by identifying areas with the greatest need for intervention and by streamlining workflows in healthcare facilities (7). By improving the efficiency and accessibility of healthcare services, AI has the potential to make high-quality cancer care more widely available across the continent, thereby reducing disparities in healthcare outcomes (15).

To fully realize the potential of AI in Type II EC care, African policymakers need to create a supportive environment for AI integration in healthcare (7). This includes investing in the necessary technological infrastructure, such as high-speed internet and data storage systems, to support AI applications (28). Policymakers should also promote the development of local AI talent by funding education and training programs that equip healthcare professionals with the skills needed to implement and manage AI technologies (27). Furthermore, there is a need for regulatory frameworks that ensure the safe and ethical use of AI in healthcare. This includes guidelines on data privacy, security, and patient consent, as well as standards for the validation and approval of AI algorithms used in clinical settings (30). Policymakers should also encourage public-private partnerships that bring together government agencies, academic institutions, and industry stakeholders to drive innovation and scale AI solutions across the continent (13).

Sustainable AI development in African healthcare systems requires a multi-faceted approach. One key strategy is the establishment of regional centers of excellence that focus on AI research and development in healthcare (13). These centers can serve as hubs for innovation, providing the resources and expertise needed to develop AI solutions that are tailored to the needs of African populations (28). Another important strategy is to foster collaboration between African countries to share knowledge, resources, and best practices in AI development (27). This can be achieved through the creation of pan-African networks and initiatives that promote cross-border cooperation in AI research and implementation (19). Finally, it is crucial to involve local communities in the development and deployment of AI technologies to ensure that these solutions are culturally appropriate and meet the needs of the populations they are intended to serve (27). This includes engaging patients, healthcare providers, and other stakeholders in the design and evaluation of AI tools, as well as ensuring that the benefits of AI-driven healthcare are equitably distributed across society (20).

Thus, the future of AI in Type II EC care in Africa is promising, with emerging trends and innovations offering new possibilities for improving diagnosis, treatment, and survival rates (9). By addressing the challenges related to technological infrastructure, data availability, and ethical considerations, and by implementing supportive policies and sustainable development strategies, Africa can harness the power of AI to transform cancer care and improve health outcomes across the continent (2). A critical part of this transformation involves establishing legal frameworks for data protection. The European Union’s General Data Protection Regulation (GDPR) offers a comprehensive standard for safeguarding personal health information in AI-driven systems. In the African context, South Africa’s Protection of Personal Information Act (POPIA) provides similar protections, setting legal standards for consent, transparency, and responsible data use. Embedding these frameworks into national policies will be essential for building public trust in AI and ensuring ethical implementation.

## 8 Sustainable development goals in AI-powered advances in type II EC

**Figure 5:**
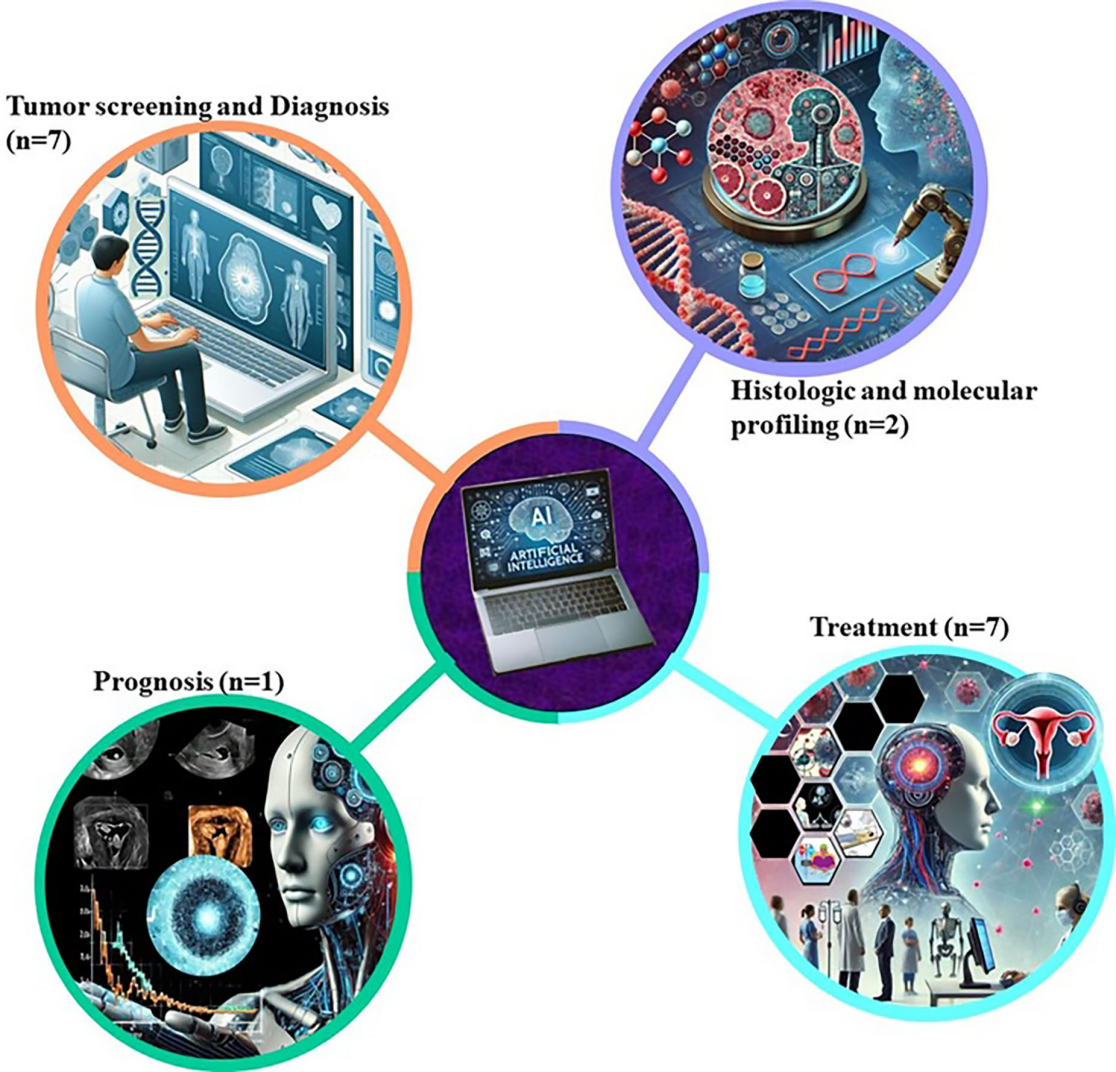
Sustainable Development Goals in AI-Powered Advances in Type II Endometrial Cancer. AI-powered advancements hold transformative potential for addressing the challenges of Type II endometrial cancer in Africa. By aligning with the SDGs, these technologies not only contribute to better health outcomes for patients but also drive sustainable development, reduce inequality, and promote gender equality and economic growth. In Africa, where resource constraints and health disparities are significant, AI offers a promising approach to advancing cancer care, with the potential to reshape the landscape of oncology through collaborative, data-driven, and patient-centered innovations.

AI-powered advancements in EC care, particularly Type II EC,align with and benefit several Sustainable Development Goals (SDGs), particularly in the context of global trends and the unique challenges faced in Africa (32). By aligning with the SDGs, these technologies not only contribute to better health outcomes for patients but also drive sustainable development, reduce inequality, and promote gender equality and economic growth (33). In Africa, where resource constraints and health disparities are significant, AI offers a promising approach to advancing cancer care, with the potential to reshape the landscape of oncology through collaborative, data-driven, and patient-centered innovations (34). AI-powered tools can analyze vast amounts of data from medical imaging, genomics, and pathology to identify unique patterns associated with Type II EC. These tools can help clinicians detect these aggressive cancers at earlier stages and develop precision treatments tailored to individual patients’ genetic profiles (32). As shown in **Figure 5**, leveraging AI can enhance early diagnosis, optimize treatment strategies, and improve patient outcomes, which supports SDGs related to health, innovation, equity, and sustainable development.

SDG 3 (Good Health and Well-being): By leveraging AI, clinicians can detect these aggressive cancers at earlier stages and develop tailored therapies which will significantly improve survival rates for patients with Type II EC.This directly supports SDG 3’s targets to reduce premature mortality from non-communicable diseases and increase access to essential healthcare services (35). In Africa, where healthcare infrastructure may be limited, AI can help predict high-risk populations based on lifestyle, environment, and genetic factors unique to the region. AI-driven risk assessment tools can guide preventive strategies and resource allocation in health systems with limited capacity, aiding the attainment of universal health coverage (33).

SDG 5 (Gender Equality): Since endometrial cancer affects women exclusively, AI advancements in diagnosing and treating Type II EC contribute to better health outcomes for women. Disparities in cancer care access are particularly pronounced in low-resource settings across Africa (35). AI tools make screening and diagnostics more accessible, helping to bridge gaps in cancer care access, aligning with SDG 5’s goals to eliminate gender-based health disparities and improve access to quality healthcare for women (34). Implementing AI in healthcare can foster new roles for women in science, technology, engineering, and mathematics (STEM) fields, particularly in healthcare AI development, medical research, and diagnostics (35). Expanding opportunities for women in these sectors supports gender equality (SDG 5’s goals) by increasing female representation in both healthcare and technological innovation (33).

SDG 9 (Industry, Innovation and Infrastructure): AI-powered cancer diagnostics and treatment models require robust infrastructure, such as high-speed internet, cloud computing, and data storage, which are often lacking in many African countries (35). Investments in digital health infrastructure to support AI applications in Type II EC not only enhance local healthcare but also drive broader technological and industrial growth which aligns with SDG 9’s goals (32). Collaborative projects between governments, healthcare providers, and tech companies can help build and maintain infrastructure that supports both healthcare and economic development in the region (13). AI offers new opportunities for research on Type II EC, particularly in understanding the genetic and environmental factors contributing to its aggressive nature (33). Africa, with its diverse population and unique environmental factors, presents a valuable context for studying these cancers. AI can facilitate studies on population-specific cancer profiles, leading to globally relevant insights that can drive innovation and improve treatment strategies for Type II EC (34). The burden of Type II EC is especially severe in regions where healthcare disparities limit access to early detection and treatment. AI can make diagnostics and treatment planning more affordable and accessible by reducing the need for expensive and highly specialized resources (34). For instance, AI-driven diagnostic tools for histopathological images can operate remotely, making high-quality diagnostics available to communities without easy access to specialized oncologists (32).

SDG 10 (Reduced Inequalities): The majority of clinical data for AI algorithms is derived from non-African populations, which can lead to biases and reduce the effectiveness of AI applications in African contexts (35). By incorporating African-specific genetic and epidemiological data, researchers can develop more equitable AI models that provide effective care for African patients, addressing inequities and improving health outcomes across diverse populations (32).

SDG 17 (Partnerships for the Goals): Advancing AI-powered solutions for Type II EC in Africa requires collaboration between governments, international health organizations, research institutions, and private sector stakeholders, thereby supporting SDG 17’s goals on partnership (34). Through partnerships, international organisations can play a crucial role in assisting AI integration in Africa. This can be done by providing support in key several areas, namely through investment in technological infrastructure, capacity building through targeted training programs, development of local AI talent while developing Afrocentric regulatory frameworks. By leveraging the strengths of both the public and private sectors, these partnerships can drive sustainable development across the continent (35). Developing AI solutions for cancer care globally relies on extensive data sharing to enhance model accuracy and relevance (35). Partnerships can facilitate global data sharing while ensuring patient privacy and ethical standards, leading to better, more inclusive AI models that benefit patients worldwide (32). Collaborative efforts can also drive policy development to regulate AI ethically and transparently, ensuring that all regions, including Africa, benefit from technological advances in cancer care (34).

## 9 Conclusion

The integration of AI into Type II EC care presents both significant opportunities and challenges, particularly within the African context. Currently, AI technologies are transforming various aspects of cancer care, including diagnostics, treatment planning, and prognosis. However, Africa faces several challenges in fully harnessing these innovations. The technological infrastructure in many regions is underdeveloped, limiting the widespread adoption of AI tools. Additionally, the availability and quality of data needed to train AI models are often inadequate, which can lead to biases and less effective solutions for African populations. Ethical and legal considerations, such as data privacy and the need for robust regulatory frameworks, further complicate the implementation of AI in healthcare. Despite these challenges, there are numerous opportunities for progress. African innovations and contributions in AI, including context-specific tools and the integration of indigenous knowledge, are promising avenues for advancing cancer care on the continent. To realize the potential of AI in Type II EC care, there is an urgent need for continued research, investment, and collaboration. Governments, academic institutions, healthcare providers, and the private sector must work together to develop the necessary infrastructure and frameworks that support AI adoption. Investment in AI research and development, particularly in the creation of datasets that accurately reflect the diversity of African populations, is essential. Collaboration between African countries and with global partners can accelerate the development of AI solutions that are effective and equitable. It is also critical to ensure that AI technologies are inclusive and serve all communities, reducing rather than exacerbating existing healthcare disparities. AI advancements in Type II EC care not only contribute to better healthcare outcomes for patients but also drive sustainable development, reduce inequality, promote gender equality and economic growth. By aligning with the SDGs, these technologies offer a promising approach to advancing cancer care and reshaping the landscape of oncology in Africa, through collaborative, data-driven and patient centered innovations.

Looking ahead, the potential of AI to transform EC care in Africa and beyond is immense. By addressing current challenges and leveraging existing opportunities, AI can revolutionize the way Type II EC is diagnosed, treated, and managed. AI-driven innovations can lead to earlier diagnoses, more personalized treatment plans, and improved survival rates, ultimately enhancing patient outcomes. In Africa, where healthcare resources are often limited, AI offers a pathway to more equitable and effective cancer care. With the right investments and policies, AI can play a pivotal role in reducing the burden of Type II EC and improving the overall quality of healthcare across the continent. The future of AI in healthcare is bright, and its successful implementation in Africa could serve as a model for other low-resource settings globally, ensuring that advances in medical technology benefit all populations, regardless of geography or socioeconomic status.
